# Causal Inference and Explaining Away in a Spiking Network

**DOI:** 10.1038/srep17531

**Published:** 2015-12-01

**Authors:** Rubén Moreno-Bote, Jan Drugowitsch

**Affiliations:** 1 Department of Technologies of Information and Communication, University Pompeu Fabra, 08018 Barcelona, Spain; 2 Serra Húnter Fellow Programme, 08018, Barcelona, Spain; 3 Centro de Investigación Biomédica en Red de Salud Mental (CIBERSAM), 08018, Barcelona, Spain; 4 Department of Basic Neuroscience, University of Geneva, Switzerland

## Abstract

While the brain uses spiking neurons for communication, theoretical research on brain computations has mostly focused on non-spiking networks. The nature of spike-based algorithms that achieve complex computations, such as object probabilistic inference, is largely unknown. Here we demonstrate that a family of high-dimensional quadratic optimization problems with non-negativity constraints can be solved exactly and efficiently by a network of spiking neurons. The network naturally imposes the non-negativity of causal contributions that is fundamental to causal inference, and uses simple operations, such as linear synapses with realistic time constants, and neural spike generation and reset non-linearities. The network infers the set of most likely causes from an observation using explaining away, which is dynamically implemented by spike-based, tuned inhibition. The algorithm performs remarkably well even when the network intrinsically generates variable spike trains, the timing of spikes is scrambled by external sources of noise, or the network is mistuned. This type of network might underlie tasks such as odor identification and classification.

The brain must efficiently implement causal inference to solve problems such as object recognition because the number of potential sensory stimuli is enormous, and also because stimuli belonging to different classes are often remarkably similar^1^,^2^,^3^,^4^. For example, we can distinguish the smell of coffee from tea and from many other similar odors with seeming ease. Yet, in terms of the computations involved, this task is hard as there are many possible odors to be recognized: if odors were made of just three chemicals out of one hundred, there would be close to one million odors among which to choose.

Massively parallel networks of relatively simple elements, such as cortical neuronal networks, are very well-suited to perform causal inference in the high-dimensional spaces that characterize human sensory domains^5^,^6^,^7^. Although hallmark network architectures have been designed that address causal inference as well as other hard computational problems^5^,^8^,^9^, these systems are based on binary or rate-based implementations, and thus do not feature the spike-based dynamics that characterize biological neuronal networks. More recent work has considered the computations that biophysically plausible spike-based networks can perform^10^,^11^,^12^,^13^,^14^,^15^,^16^, including learning and solving causal inference problems, and typically, but not always, using stochastically spiking units^17^,^18^,^19^,^20^. Despite this progress, it still remains unknown in general what causal inference problems can be solved by biologically realistic spiking networks.

In this paper we show how and under which conditions spiking networks can perform causal inference over high-dimensional spaces that is representable as a quadratic programming problem with non-negative inequality constraints. We demonstrate that a specifically tuned network of integrate-and-fire neurons can compute the set of most likely causes given a noisy observation of a linear combination of these causes weighted by non-negative coefficients. This requires finding the solution to a high-dimensional quadratic optimization problem with non-negativity constraints, an operation that cannot be achieved by linear networks. As expected, our networks find the solution using explaining away, which suppresses irrelevant causes when the observation can be already explained^2^,^3^. The novelty of our implementation is that, rather than providing a rate-based solution, explaining away operates dynamically solely by the use of spikes. The network design is remarkably simple, consisting of linear synaptic interactions with realistic time constants and hard non-linearities for the spike generation mechanism. Despite such a straight-forward implementation, the spiking network can discriminate a cause among a multitude of other similar causes in just a few spikes. When confronted with a complex mixture of causes, the network can exactly and efficiently determine all the components of the mixture. The network is robust against internally and externally generated sources of variability, and against mistuning in the recurrent connectivity. We show that, rather than encoding information about the causes in the firing rates of individual neurons or in precise spike timing, this information is encoded in the slow covariations of the firing rates of the whole neural population.

The dynamics and architecture of our networks are closely related to other rate-based and spiking networks for stimulus representation, stimulus tracking and approximate quadratic programming^13^,^14^,^21^,^22^,^23^, but depart from previous work in the specific details of derivation and neuronal architecture and in the use of more flexible synaptic kernels. Furthermore, the dynamics of our networks substantially differ from networks based on sampling of probability distributions^17^,^24^,^25^ and goes beyond cue combination and marginalization in lower-dimensional spaces^12^,^26^ by focusing on higher-dimensional inference problems.

## Results

### Generative model of causes and observables

Many every-day decisions are based on inferences about hidden causes from ambiguous and noisy observations. Consider inferring the cause of observing a wet pavement in the morning. Many events could have caused it being wet, such as rain during the night, or a gardener watering a park nearby. In many other related common tasks, like in object recognition, such inference problems become increasingly difficult: a very large set of causes can originate from very similar observations, such as, for example, when trying to identify a person from the contours of her shadow. Our aim is to study the algorithms that can be implemented by spiking networks to solve Bayesian inference problems like those above.

**Figure 1:**
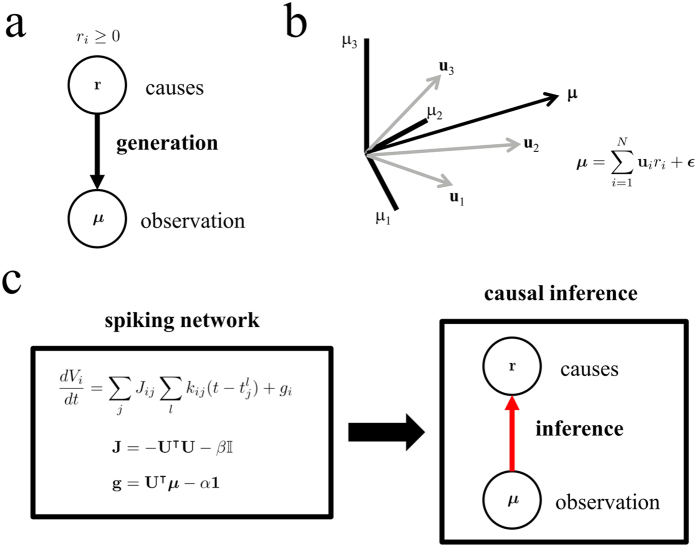
A spiking network can exactly solve a high-dimensional causal inference problem. (**a**,**b**) Generative model. A potentially very large number *N* of hidden causes generate an observation (**a**). Each cause *i* is represented as an entry of the *N* dimensional vector ***r***, and it is characterized by a non-negative number, *r*_*i*_ ≥ 0, called cause coefficient. The cause coefficient *r*_*i*_ indicates both presence of cause *i*, if non-zero, and its strength, such as contrast or concentration. Associated to each cause *i* there is a feature vector **u**_*i*_ of dimension *M*. The observation ***μ*** is a linear combination of the feature vectors –causes– weighted by non-negative cause coefficients *r*_*i*_ and corrupted by noise (**b**). (**c**) A network of integrate-and-fire neurons with tuned inhibition implements dynamic, spike-based explaining away and solves a causal inference problem corresponding to quadratic programming with non-negativity constraints. Global inhibition (*α* term) and renormalized reset voltages (*β* term) implement L1 and L2 regularization, respectively.

We consider a high-dimensional inference problem where an arbitrary combination of *N* causes can generate an observation (Fig. 1a). The observation is described by an “input” vector ***μ*** of dimension *M* (e.g., gray levels of an image with *M* pixels, or *M*-dimensional chemical composition of an odor), which is generated as a linear combination of causes corrupted by noise,


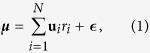


where **u**_*i*_, *i* = 1, ..., *N*, are the “feature” vectors, which correspond to causes *i* = 1, ..., *N* respectively (Fig. 1b) (We typically reserve bold lower-case letters for column vectors and bold capital letters for matrices). We assume that the feature vectors have been learned and are thus known. The *i*^*th*^ feature vector can be viewed as the representation of the *i*^*th*^ cause (e.g., Gabor functions for an image, or the collection of chemicals with their relative concentrations that constitutes an odor percept). The feature vectors **u**_*i*_ have length *M*, but the number of them, *N*, can be a priori much larger than *M*. The feature vectors are weighted by the cause coefficients {*r*_*i*_}, *i* = 1, ..., *N*, which are strictly non-negative, *r*_*i*_ ≥ 0. Additionally, the linear combination of features with non-negative coefficients is corrupted by independent Gaussian noise, 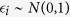.

The cause coefficients {*r*_*i*_} are assumed to be non-negative to enforce the intuition that causes can be either present or not present, but they cannot be “negative”; namely, we do not consider causes as sinks for other causes, but we rather see them as quasi-independent objects in the world. In our setup, if the *i*^*th*^ cause is not present, then the *i*^*th*^ cause coefficient is zero, *r*_*i*_ = 0. If the *i*^*th*^ cause is present, then its cause coefficient *r*_*i*_ becomes positive. The additional degree of freedom allowed for the positiveness of *r*_*i*_ in the last case permits the encoding not only of the presence of the cause, but also its intensity. In other words, the larger the value of *r*_*i*_, the larger the intensity of cause *i* (e.g., the contrast of an object in the image, or the concentration of an odor). Both the non-negativity constraint of the coefficients and the linearity of the combination of the causes in Eq. (1) are well-justified in several relevant examples: (i) an image such as a face can be approximately described by a superposition of complex features, such as the color of the eyes, the shape of the nose, and so on^21^, and (ii) an odor is made of a positively-weighted superposition of more basic odorants^27^. As a concrete example, consider our internal representation of an odor mixture, like the one that we can smell during breakfast, as a combination of stored basic odors, such as coffee and pancakes. In this case, the input vector ***μ*** represents the input odor mixture, and the feature vectors **u**_*i*_ represent more basic complex odor objects, such as coffee, pancakes, and so on. The non-negativity of the weighting coefficients *r*_*i*_ enters naturally in this kind of problems because it is unconventional to describe any odor mixture as having negative contributions.

We assume that a priori, that is, in the absence of any observation, causes in the world are independent and rare, and take non-negative values following a truncated Gaussian


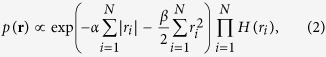


where *α* ≥ 0, *β* ≥ 0 and *H*(*x*) is the Heaviside function (*H*(*x*) = 1 if *x* > 0, and *H*(*x*) = 0 otherwise). The latter ensures that cause coefficients can never be negative. This prior distribution over the causes includes as a special cases the Laplace prior (*β* = 0) and the truncated Gaussian centered at zero (*α* = 0).

Given the generative model for the input vector, Eq. (1), and the prior over the causes, Eq. (2), the joint probability distribution for both of them is


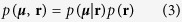



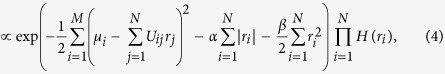


where *U*_*ij*_ are the entries of the *M* × *N* matrix **U** = (**u**_1_, **u**_2_, .., **u**_*N*_), whose *j*^th^ column is the vector **u**_*j*_. Using matrix notation, the joint probability distribution can be rewritten as


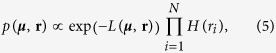


where we have defined the negative log-posterior function


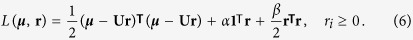


Here, **1** is a vector with all entries equal to one, and superscript T denotes the transpose operator.

Upon observing the input vector ***μ*** we want to use the generative model described above (Fig. 1a,b) to infer which were the causes that generated this input. The goal, therefore, is to find the minimum of the function *L*(***μ***, **r**) for a given ***μ***. Discovering the cause coefficients **r** that minimizes this function corresponds to the maximum a-posteriori (MAP) estimate of **r**. Minimizing Eq. (6) with the non-negative constraint makes the problem non-linear, such that a simple matrix inversion is not sufficient to determine the optimal solution. This problem is also equivalent to a sub-class of quadratic programming problems with inequality constraints^28^.

Minimizing Eq. (6) is akin to finding a good approximation of the input vector as a linear combination of features with non-negative values,


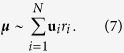


While the first term in the r.h.s. of Eq. (6) penalizes large differences between the input vector and this linear approximation, the second and third terms penalize large values of the coefficients *r*_*j*_ *per se*. The last two terms can also be interpreted as L1 and L2 regularization terms, where *α* and *β* are the penalty coefficients.

Expanding quadratic terms in Eq. (6) and defining 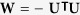 and 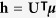, it is easy to verify that minimizing Eq. (6) over **r** at fixed ***μ*** is equivalent to minimizing the energy function


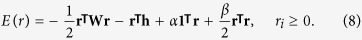


If the feature vectors are linearly independent, then 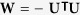 is negative definite, and as a consequence the energy has an single minimum in the convex set *r*_*i*_ ≥ 0, *i* = 1, ..., *N*. If the feature vectors are linearly dependent, then **W** is negative semi-definite. In this case, the energy still has a single minimum if either *α* > 0 or *β* > 0, and otherwise multiple states **r** associated with the same minimal energy.

### Solving the causal inference problem with a rate-based network

There exist various efficient algorithms^28^ to minimize the loss function in Eq. (8) (or equivalently Eq. (6)). Here we build a single-layer rate-based network that minimizes this energy with non-negative firing rates (see [21] for a two-layer network implementation). In this network, as well as in the spiking network described in the next section, the number of neurons is usually designed to match the number of causes to be represented in the world. This is not a severe restriction on the neuronal architecture, as the network can easily incorporate situations in which hidden causes are overrepresented (i.e. encoded by a combination of multiple neurons) by selecting identical or very similar feature vectors for some neurons. In either case we assume that the rate *r*_*i*_ of the *i*^*th*^ neuron in the network follows the dynamics


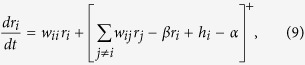


where *w*_*ij*_ = (**W**)_*ij*_, **W** is as defined in Eq. (8) and [*x*]^+^ is the linear rectified function ([*x*]^+^ = *x* if *x* ≥ 0, and [*x*]^+^ = 0 otherwise). Like in many other functional networks, the connectivity **W** is symmetric^5^,^9^, although we show below that this assumption is not critical. The linear rectification enforces the constraint that rates are non-negative. This is because if the initial condition of the rates is in the non-negative orthant (*r*_*i*_ ≥ 0 for all *i*), then the trajectories remain confined to that region. We abuse notation by using **r** for both the firing rates of the network in this section and for the cause coefficients in the previous sections. The idea is that the firing rate of the network at the fixpoint corresponds to the most likely causes in Eq. (8), as shown next. We only consider the case in which the energy in Eq. (8) has a unique absolute minimum, albeit not necessarily in the non-negative orthant.

It can be shown that the dynamics defined in Eq. (9) acts to reduce the energy Eq. (8)


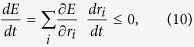


for all **r**, and therefore performs gradient descent over the objective function^29^. Equality to zero only holds for the (unique) minimum of the energy restricted in the non-negative orthant. This implies that the dynamics in Eq. (9) approaches the minimum of the energy in Eq. (8) restricted in the non-negative orthant. This minimum, **r**^MAP^, which corresponds to the MAP estimate of the cause coefficients given the observation, obeys the system of non-linear equations


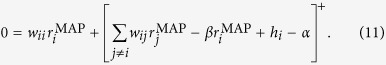


This result will be important to show in the next section that a specific spiking network can minimize Eq. (8).

### Solving the causal inference problem with a spike-based network

Based on the results derived for the rate-based network, in this section we build a network of integrate-and-fire neurons that solves the quadratic programming problem with non-negativity constraints defined in Eq. (8). Previous work has dealt with a similar quadratic programming problem^23^ in a network of explicitly leaky neurons, to derive a greedy, dynamic solution, based on an argument of dynamic loss minimization. We, instead, use a different approach to derive the exact steady-state solution for our problem in the low-leak regime. We first consider a network of *N* leaky integrate-and-fire neurons with arbitrary network connectivity, and later tune it to perform causal inference in our problem. The membrane voltage *V*_*i*_ of neuron *i* in the network follows


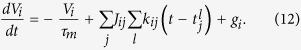


Here, *τ*_*m*_ is the membrane time constant of the neuron, *J*_*ij*_ is the connection strength between pre-synaptic neuron *j* and post-synaptic neuron *i*, 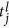 is the time of the *l*^th^ spike of neuron *j*, 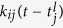 is the synaptic response to that spike and *g*_*i*_ is the input synaptic drive.

Neuron *i* emits a spike whenever its voltage reaches a threshold value Θ, after which the voltage is reset to a hyperpolarized value *H*_*i*_ < Θ. This reset is implemented by a self-inhibitory current


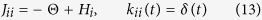


in Eq. (12), where *δ*(*t*) is the delta-function. In other words, a spike of neuron *i* causes its voltage to instantaneously drop from the threshold value Θ to the reset value *H*_*i*_.

For *i* different from *j*, the synaptic kernel *k*_*ij*_(*t*) corresponds to a brief synaptic current in neuron *i* generated by a spike from neuron *j*. This kernel is zero when *t* < 0 and, for convenience, we assume that it integrates to one,


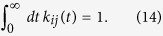


Although our results are valid for arbitrary kernels, we typically use exponential synaptic kernels *k*_*ij*_(*t*) = exp(−*t*/*τ*_*s*_)/*τ*_*s*_, for *t* > 0 with realistic time constants of 3–10 ms.

When the input drive is large, the leak term is dominated by both external and recurrent inputs, and therefore the dynamics can be well approximated by a network of non-leaky integrate-and-fire (nLIF) neurons,


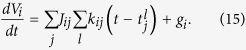


Such large input drives are expected to occur when the network is in the balanced regime, and/or when external inputs to the networks are large and supra-threshold. For this network, we are interested in determining the firing rate for each neuron in the long run. To compute these firing rates, we first integrate both sides of Eq. (15) from 0 to *T* to obtain^30^


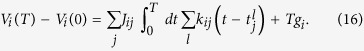


The integral involves terms that can be expressed as


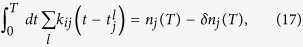


where *n*_*j*_(*T*) is the spike count of neuron *j* from time 0 up to time *T*. The residual term *δn*_*j*_(*t*) (0 ≤ *δn*_*j*_(*t*) ≤ 1) arises from the fact that *k*_*ij*_(*t*) has finite width in time (if *i* ≠ *j*).

For long enough integration window *T*, the terms in Eq. (16) scale differently with time, and this scaling depends on the firing state of the cell. If, on one hand, neuron *i* is active in the long run, then the term *V*_*i*_(*T*) − *V*_*i*_(0) is *O*(1), while the two terms in the r.h.s. scale with *O*(*T*). If, on the other hand, neuron *i* is inactive, then the term *V*_*i*_(*T*) − *V*_*i*_(0) is *O*(*T*) and negative because a net inhibitory current into the cells causes its voltage to drift to very negative values. These two sets of conditions can be combined into a self-consistency system of equations for the time-averaged firing rates (defined as 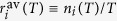)^31^,


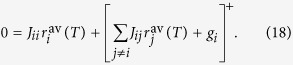


This equation states that if the total average input current into a neuron (argument of the rectified function) is negative, then its firing rate (i.e., spike count over a long time) is zero. If the input current is positive, then the firing rate is positive (note that *J*_*ii*_ is negative). The linear rectification function guarantees that these two conditions hold simultaneously.

Now we can see that the self-consistency equation for the time-averaged firing rates of the spike-based network, Eq. (18), is identical to the unique solution of the firing rate in the rate-based network, Eq. (11), if the connectivity matrix **J** and external currents **g** of the nLIF network in Eq. (15) are set as


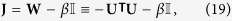



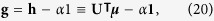


where 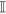 is the identity matrix. The connectivity matrix specifies both the neuron-to-neuron connectivity as well as the reset voltage for each neuron. Specifically, the off-diagonal entries of **J** determine the neuron to neuron connectivity, while the diagonal entries *J*_*ii*_ determine the reset voltage. More precisely, the relationship between the reset voltage of neuron *i* and *J*_*ii*_ is given by


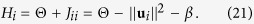


It is noteworthy that the effect of L1 and L2 regularization is to introduce a global inhibitory term proportional to *α* (Eq. (20)) and to lower the reset voltage by *β* (Eq. (21)), respectively. Therefore, the roles of L1 and L2 regularization are different in terms of their expected effects on population activity: while L1 makes responses sparser, L2 lowers responses uniformly without changing sparsity, as shown later.

With the above choices the time-averaged firing rates of the network obey the equation


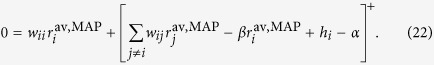


This equation is identical to Eq. (11), which corresponds to the minimum of the energy in Eq. (8) in the non-negative orthant. Therefore, the time-averaged firing rates of the spiking network defined in Eqs. (8)–(19)–(20) represent the MAP causes given the observation (Fig. 1c). Although the theory that we have described only applies exactly to nLIF networks, we will show through simulations below that LIF networks behave similar to nLIF neurons with *α* > 0^32^.

For the particular case in which the synaptic kernels are delta-functions, the spiking network follows the dynamics


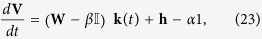


where **V** is the vector of the neurons’ membrane voltages, and 
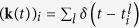
. For analytical continuity reasons, the delta-functions should be understood in the following sense: an excitatory input spike from neuron *j* to neuron *i* depolarizes the voltage of the latter by an amount *J*_*ij*_, and if this quantity exceeds threshold the excess is added to the reset voltage after the spike. Finally, note that although using delta-functions as synaptic kernels simplifies notation, they are not necessary to minimize Eq. (8), and we typically use exponential synaptic kernels with realistic time constants of 3–10 *ms* in most of our numerical examples.

### Dynamic, spike-based explaining away as underlying algorithm

We have shown that a tuned nLIF network can solve a high-dimensional causal inference problem that corresponds to quadratic programming with non-negativity inequality constraints. Can we dissect the dynamics of the network and understand what is the underlying algorithm used to solve this problem? Not surprisingly, we find that the network solves the minimization problem in Eq. (8) by explaining away, implemented dynamically through spikes. To see how this works, first we rewrite the time-averaged firing rate for neuron *i* in Eq. (22) as


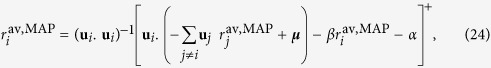


where we have used *J*_*ij*_ = −**u**_*i*_.**u**_*j*_ − *βδ*_*ij*_ and grouped terms proportional to **u**_*i*_. The term within the inner brackets is the reconstruction error of the input vector ***μ*** based alone on the activity of all the neurons except neuron *i*. If this error is zero, then the firing rate of neuron *i* would be zero: that is, the activity of other neurons ‘explains away’ the stimulus and there is no need to recruit the activity of new neurons to approximate the stimulus. If the error is non-zero, then the firing rate of neuron *i* is approximately the projection of the error onto the feature vector **u**_*i*_, that is, proportional to how similar the error is to the feature vector encoded by neuron *i*. The network performs these operations dynamically through specifically spike-based tuned inhibitory interactions until the optimal solution is found.

### Accurate and rapid causal inference in demanding tasks

**Figure 2:**
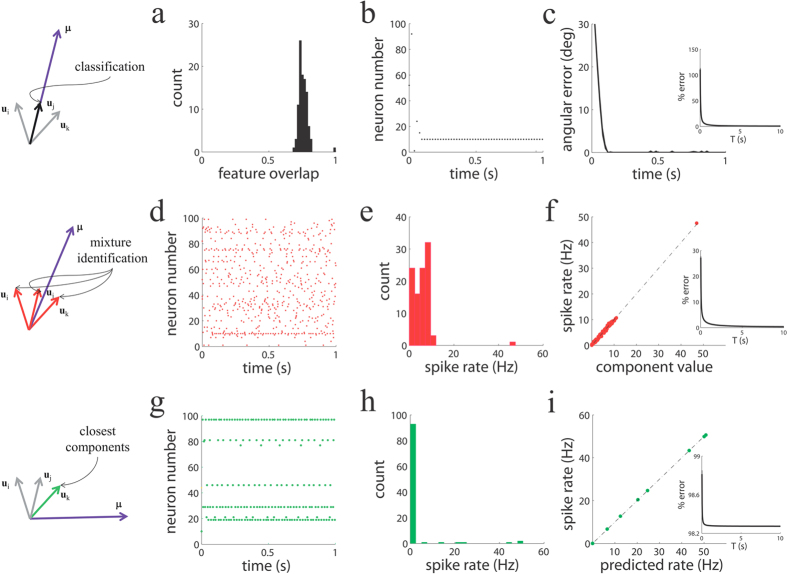
The spiking network solves efficiently a hard discrimination task in a few spikes (top row), identifies all the components of a complex mixture (middle) and finds the closest causes to an odd observation (bottom). Schematics for the three tasks are shown on the left. The causes that need to be identified are marked with the same color than the panels on their right. For instance, in the classification task, the input vector is proportional to feature vector *j* (*j* = 10) and this is the cause that has to be identified. (**a**) Distribution of overlaps between feature vectors, centered at around 0.75 (Methods). (**b**) Response of the spiking network upon stimulation with the 10^*th*^ cause. Only the neuron that encodes the cause used as a input (the 10^*th*^ neuron) is active after a brief transient consisting of a handful of spikes from other neurons. (**c**) Angular error decays to close to zero in about 100 *ms* (*T* = 20 ms time windows). Inset: percentage error (see Methods) decays to zero as the integration window *T* increases. (**d**,**g**) Population activity of the network stimulated by a strong cause buried under a strong random background (**d**) or by an input vector that cannot be exactly represented by the feature vectors (**g**). (**e**,**h**) Distribution of firing rates. (**f**) Observed firing rate of the spiking network vs. the components of the mixture. The spiking network finds the mixture of features that composes the input vector. Inset: percentage error decays to zero as integration window *T* increases. (**i**) Observed firing rate of the spiking network vs. the rate predicted from a non-spiking algorithm for the same problem (rate-based network algorithm) in the approximation task. Inset: percentage error decays to the minimum (optimum) value as integration window *T* increases. In this case the error does not approach zero because in the approximation task the input vector is outside the convex hull formed by the set of all feature vectors.

The theory presented above does not specify how quickly and robustly our spiking networks reach the steady state. In particular, the presence of very slow transients could make convergence to the fixpoint extremely slow. Therefore, it is important to test their performance in a few relevant demanding tasks. While, in principle, truly different tasks would involve changing and learning the prior distribution over the causes, here we simply define task as a specific type of input configuration while keeping fixed the prior distribution over causes. We show in this section that our spiking network can exactly (1) discriminate a cause among a multitude of others with just a handful of spikes, (2) identify the components of a complex mixture, and (3) approximate an odd input vector. We first test whether the network can identify a single cause out of many potential causes (one hundred potential causes, *N* = 100), and how fast it can do so. In addition to making the task high-dimensional, we made it difficult. This was accomplished by generating very similar causes, such that their associated feature vectors were strongly overlapping and therefore difficult to distinguish (Fig. 2a, average overlap ~0.75; Methods). Despite the high-dimensionality of the space of causes and the high similarity, the network was able to detect the presence of the correct cause: only the neuron that corresponded to the cause used as a stimulus was consistently active over time (Fig. 2b). As a result, the response of the network is very sparse, with just one neuron being active after a brief transient response. Importantly, convergence takes only a small number of spikes. On average, the network converges rapidly to the correct solution with a time scale smaller than 100 *ms* (Fig. 2c), corresponding to just a handful of spikes from neurons coding for other causes.

One potential caveat of our networks is that, after convergence to the correct solution, the voltage of inactive neurons becomes very large and negative due to the non-leaky nature of the network. This can be corrected by making cells adaptive by introducing a leak term whenever the stimulus intensity is below some criteria. Such an adaptive leak would support the recovery of the population activity to voltages close to reset values within stimulation periods.

Next, we study whether the spiking network can correctly identify all the components of a complex mixture of causes. The task is made difficult by combining a strong cause with a strong random combination of the remaining causes. This situation can correspond to the case in which an odorant, such as coffee (strong cause), is mixed with many other weak odorants, such as those arising from the cafe where we are having breakfast (strong background). In this case, spiking activity is distributed among the population of neurons because causes encoded by many neurons are recruited to explain the stimulus mixture (Fig. 2d,e). In particular, the neuron that encodes the strong cause fires at the highest rate (Fig. 2d). We also confirmed that the solution attained by the spiking network corresponded to the actual mixture used (Fig. 2f) and that the solution converges with an increasing integration window (inset).

In a task in which the input vector does not correspond to any non-negative sum of the features vectors, the network must approximate the odd input to the closest features. In this approximation task, the activity of the network is sparse, with only a bunch of neurons being active throughout the stimulation period (Fig. 2g,h). We confirmed that the solution attained by the spiking network corresponds to the optimal solution, as the network delivers the same set of approximating causes as a non-spiking algorithm for the same problem (Fig. 2i; see rate-based algorithm in Eq. (9)) and the solution converges to the minimum error with an increasing integration window (inset).

**Figure 3:**
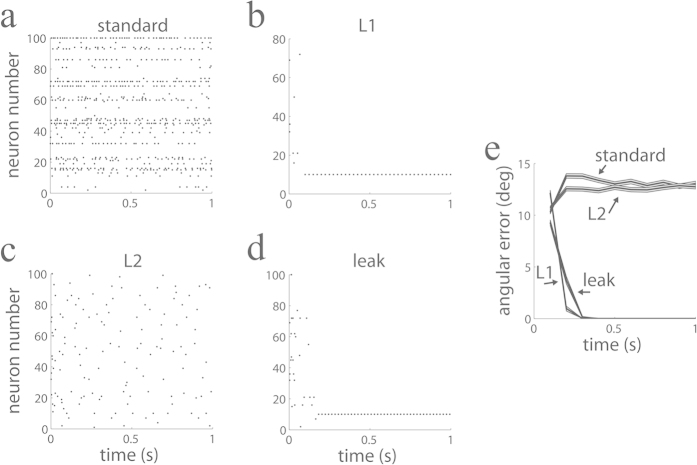
Effects of regularization on population activity and performance in a network with an overcomplete basis of feature vectors. (**a**–**d**) Spiking activity for the standard network without regularization (**a**), with L1 regularization (**b**), L2 regularization (**c**), and voltage leak (**d**). In all cases input equals the 10^*th*^ feature vector. (**e**) Angular error as a function of time (100 *ms* time windows).

We also compared the behavior of the network with and without regularization terms in an overcomplete scenario, namely, in a case in which the dimensionality of the input vector was smaller than the number of features (Fig. 3). We stimulated the network with a single feature and studied its identification performance. We found that L1 regularization, implemented in our networks as global inhibition, creates a sparser representation of the input vector than the same network without L1 regularization (Fig. 3a,b). With L1 regularization, the network converges to the true input vector in just a few spikes (Fig. 3b), and the angular error decays to zero in a few hundreds of milliseconds (Fig. 3e). The reason for the convergence is that in our simulations the stored feature vectors are normalized to the same length, 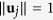. In this case, L1 regularization always produces sparse representations of the stimulus vector if the stimulus coincides with one of the stored feature vectors. If stored feature vectors had unequal lengths, then the stimulation of one stored feature would have led to non-sparse firing. To make this clear, assume that due to the overcomplete representation of the stimulus space, the feature vector **u**_*i*_ can be expressed as a sum of other feature vectors as 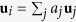, with *j* ≠ *i* and *a*_*j*_ ≥ 0. If this is the case, there are at least two distinct activity patterns that can fully represent the stimulus ***μ*** = **u**_*i*_: the first one is a sparse one that consists of a single neuron (neuron *i*) firing at rate *r*_*i*_ = 1 *Hz* and all other neurons being inactive, while the second activity pattern is a non-sparse pattern where neurons fire at rate *r*_*j*_ = *a*_*j*_ for all *j* ≠ *i*. However, given the equal normalization of all feature vectors, it is easy to see that 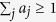, and therefore L1 regularization, which penalizes large total population activity, will favor the sparse over the dense pattern.

When L2 regularization is used instead of L1, the spiking density is reduced when compared to the un-regularized case, but the response is less sparse than with L1 regularization (Fig. 3c). Moreover, the angular error does not decrease over time (Fig. 3e). Therefore, L2 regularization, implemented in our networks in the form of a lower reset value, does not typically perform as well as L1, in the sense that in an identification task the angular error with L2 is larger than the one with L1. Similar results were found when we moved from a single-cause identification tasks to identifying mixtures of causes.

Interestingly, when instead of regularization, the spiking neurons are leaky, the network also finds correctly the input feature (Fig. 3d,e), at a speed comparable to the L1 regularized spiking network. This is because the leak term of the voltage introduces a negative current that on average is well approximated by the global inhibition characteristic of L1 regularization for the particular value *α* used. The similarity between leaky and non-leaky networks is compromised if the values of *α* are too small or large: too small *α* will lead to too weak sparsity due to weak global inhibition, while too large *α* will lead to too strong sparsity because too few neurons will fire.

### Stimulus representation is stable over time despite large spiking variability

Our algorithm for performing causal inference for the problem in question relies on spike codes. The fact that this algorithm encodes the causes in the neurons’ firing rates over the long run does not preclude the possibility that the functioning of the algorithm strongly depends on the dynamic coordination of spike timing between neurons. If our algorithm indeed relies on such a precise coordination, then its performance should be strongly compromised in the presence of spiking variability and noise, as these act on spike timing by shifting it. In the presence of large amounts of such spiking variability, as observed in cortex^33^,^34^, the situation can only be worse, thus rendering any algorithm that relies on precise spike timing useless. Therefore, it is important to test the robustness of our spiking network against the presence of (i) spiking variability intrinsically generated by the neuronal dynamic and (ii) external sources of noise.

**Figure 4:**
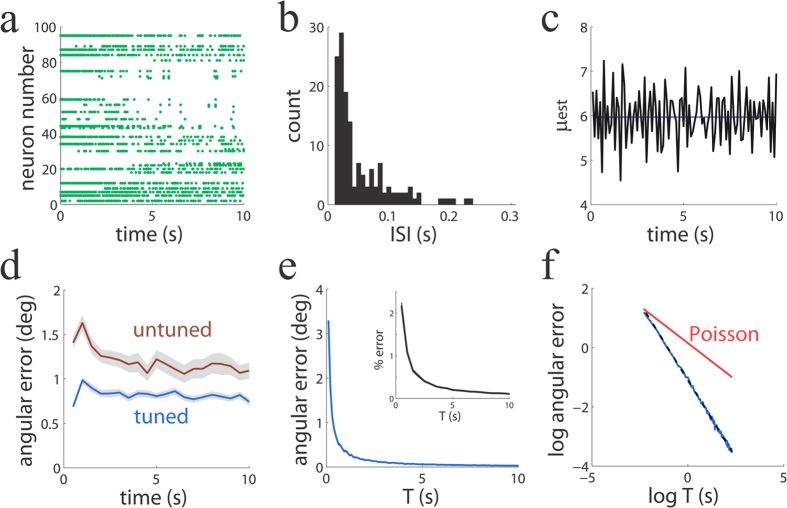
Input information is faithfully represented over time in spite of large spiking variability. (**a**) Population spiking activity over time (**b**) Distribution of ISIs of a representative neuron. (**c**) The estimate of the stimulus (jagged line) reproduces the true stimulus (blue) and is stable over time. First component of the estimate and stimulus are shown (100 ms time windows). (**d**) The angular error is stable over time for both a perfectly tuning (blue) and a mistuned (brown) network (500 ms time windows). (**e**) Angular error (inset: percentage error) as a function of the integration window *T*. (**f**) Log-log plot of the previous panel. Angular error (blue line) is tightly fit by a line with slope −1.04 (black; almost invisible as it is overlaid by the data). An ideal population of cells firing independently with Poisson statistics would produce a slope of −1/2. This prediction is plotted (red) for comparison. The left-most point for this Poisson prediction was made equal to the observed networks performance fit for visual comparison.

We first address the question of whether intrinsically generated spiking variability harms the performance of our networks. To generate spiking variability intrinsically by neuronal dynamics, we created a spiking network where the dimensionality of the stimulus was much lower than the number of neurons, *M* ≪ *N*. Because the *N* × *N* connectivity matrix **J** is a low rank matrix with rank *M* ≪ *N*, the neuronal dynamics offers a highly overcomplete representation of the input space and becomes a multi-dimensional attractor^35^. Without any regularization of the dynamics and in absence of noise, the corresponding rate-based network converges to a point on this multi-dimensional manifold attractor, determined by the initial conditions. The spike-based implementation can be interpreted as a noisy version of the rate-based network, such that the spiking network traverses the attracting manifold in a quasi-random walk, despite not having any truly stochastic component in its dynamics. In this scenario, which is specific to the overcomplete representation of inputs, the same stimulus can be faithfully represented by potentially many different activity patterns consisting of different sets of neurons being active and representing different combination of causes^13^. This representation can evolve over time, and the observed complex dynamics can be interpreted as variability. Our simulations show that, for each neuron, firing is very irregular (Fig. 4a), with a broad distribution of high inter-spike-intervals (ISI) (Fig. 4b). The population averaged coefficient of variation of the inter-spike-intervals (*CV*_*ISI*_) was *CV*_*ISI*_ = 3.20, larger than the one typically observed in sensory cortex^33^,^34^, but consistent with the larger variability found in prefrontal areas^36^. The presence of variability was robust to changes in the synaptic kernels used. When instead of using exponential kernels we used delta-function kernels with no delay or with 2 *ms* delay, the network generated high variability with population averaged *CV*_*ISI*_ = 3.48 and *CV*_*ISI*_ = 2.89, respectively. The variability observed in larger networks of up to *N* = 500 cells (*CV*_*ISI*_ = 2.98) was also comparable to the variability observed in smaller networks of *N* = 100 cells (*CV*_*ISI*_ = 3.48). Despite the sheer irregular activity in the network, the encoding of the stimulus is fairly stable over time (Fig. 4c). A relatively stable decoding error of around 1deg is attained (Fig. 4d, blue line). Therefore, the spiking network is able to represent a complex input pattern in a reliable way over time in spite of intrinsically generated spiking variability.

We also asked whether the networks behavior was robust to perturbations of the optimal connectivity described in Eq. (19). This question is important because it is possible that very small deviations of the optimal architecture might have large effects on performance. This was indeed what happened when we perturbed the optimal values of the network connectivity in Eq. (19) by adding a component with a value that was independent across contacts and uniformly distributed in the range between −0.1 and 0.1: a network with this size and type of mistuned connectivity became typically unstable because a few eigenvalues of the connectivity matrix become positive. However, this connectivity perturbation can be viewed as too strong because it destabilizes the whole network dynamics, leaving very little room for the possibility of performing any useful computation. Homeostatic and synaptic learning mechanisms exist that might aid the stability of a network^37^ and can be implemented efficiently by inhibitory synaptic plasticity^38^. Such strong inhibition, which is characteristic of balanced networks, finds experimental support^39^,^40^. Following this idea, instead of perturbing the network connectivity by zero-mean noise, we perturbed the connectivity by adding i.i.d. perturbation with a sufficiently large negative mean (−0.1; uniformly distributed in the range between −0.2 and 0) to all the entries of the connectivity matrix. In the presence of this negative bias in the unturned connectivity the network remained stable and, interestingly, featured a performance (Fig. 4d, brown line) that was very much like the tuned network (blue).

We furthermore confirmed that the performance of the mistuned network did not substantially worsen (it was actually slightly improved in relation to the brown line in Fig. 4d) when we used delta-function kernels with delay (2 ms) instead of the more realistic exponential kernels that we have used so far. Interestingly, the observed small degradation of performance in both types of mistuned networks compared to optimal networks contrasts with the large change of the values in the connectivity matrix (Eq. (19)), which incur an on average 75% change from their optimal values. Similar robustness to perturbations have been found previously in other spiking networks with comparable architecture^14^.

### The precision of irregularly spiking networks is as high as that of perfectly regular spiking networks

One important question is how precise stimulus encoding is in the presence of high spiking variability. While the angular error converges to zero in regularly spiking networks at finite integration windows (Fig. 2c), the angular error does not decay to zero in irregularly spiking networks (Fig. 4d). Is this difference real, or does it just depend on the finite size of the integration windows used to estimate firing rates? As in the analyses of Fig. 4 we were using finite windows, there is the possibility that regularly and irregularly spiking networks appear to feature different decoding errors simply because it is more difficult to reliably estimate the firing rate in small integration windows when the spiking is highly variable. If this is the case, using a larger integration window *T* to estimate these rates should make these estimates more reliable, such that both approaches can be compared on an equal footing. Even in a regularly spiking network for long integration windows *T*, a spike of the periodic cycle can be missed, resulting in an error of the firing rate estimate of the order of 1/*T*. If irregular firing in our simulations results in deterioration of information beyond that caused by unreliable rate estimate, we should observe an error that decays more slowly than 1/*T*. In particular, if firing were Poisson and independent across neurons, we would expect that the decoding angular error would scale at the slower pace 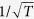 for long *T*. We computed the decoding angular error as a function of *T* (Fig. 4e) and plot it in a log-log scale to study its scaling behavior (Fig. 4f, blue line). The log error decayed linearly as a function of the log of *T* with a slope very close to −1 (−1.04 ± 0.01; 95% confidence interval), showing that the error approximates 1/*T*. When we used percentage error (see Methods) instead of angular error we also found that the error decays monotonically towards zero, as expected from our theory. Therefore, a highly variable spiking network shows a decoding performance that is as good as one would expect from a network that spikes perfectly regularly, and much better than the performance expected from Poisson-firing networks (Fig. 4f, red line). Interestingly, this seems to be a general property that has already been observed before in related computing spiking networks with comparable architecture^14^.

### Slow firing rate covariations of activity underlies reliable stimulus encoding

**Figure 5:**
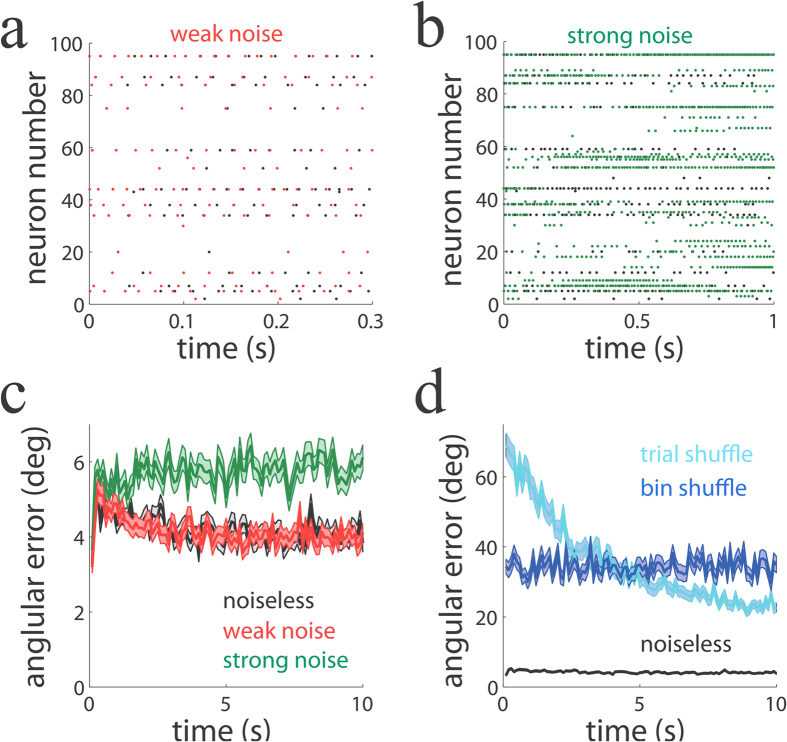
Slow firing rate covariations underlie reliable encoding. (**a**,**b**) Population activity patterns over time for a noiseless (black dots), weak-noise (red) and strong-noise (green) network. The noiseless network is identical in the two panels but represented at two different time resolutions. Networks only differ in the injected noise variance, while other parameters including initial conditions are identical (Methods). (**c**) Angular error as a function of time for the three networks (100 ms time window). (**d**) Angular error as a function of time for the noiseless network (black line) and for trial- (light blue) and bin- (dark blue) shuffled networks. When the slow covariations of firing rate are destroyed by the shuffling, performance largely deteriorates compared to the one of the noiseless network.

So far we have confirmed that our spiking network performs causal inference with high accuracy even when there are internally generated sources of variability. Now we turn to the second question: Is our spiking algorithm robust against external sources of noise? To answer this question, we compared a network with no input noise (reproduced in Fig. 5a,b, at two different time resolutions; black dots) to networks in which weak (red) or strong (green) noise was injected.

When a very small amount of noise was externally injected into the network, spike times were notably shifted compared to those of the noiseless network (Fig. 5a, black and red dots). Despite this significant spike time perturbation, there was virtually no difference between the reconstruction angular errors in the noiseless and weak-noise networks (Fig. 5c, black and red lines). When the injected noise was increased 30-fold, both spike timing and population activity patterns were remarkably different when compared to those in the noiseless network (Fig. 5b, black and green dots). However, surprisingly, there was only a modest increase in the reconstruction angular errors when compared to the noiseless network (Fig. 5b, black and green lines), which was much smaller than expected from the observed large differences in activity patterns (Fig. 5b). These results show that our spiking network is robust against external sources of noise, and seem to argue, perhaps more importantly, that precise timing does not play an important role in the encoding and functioning of the network.

If spike timing does not play an important role in the encoding of the stimulus and the underlying causes, where is the information encoded? The theory and analysis described above clearly indicates that the relevant variables for the encoding of the causes are the neurons’ firing rates (spike counts over a long integration window). But where is information encoded when the integration window is not very large? Is the coordination of the firing rates across neurons required in this case? If the coordination of firing rate is important for encoding information at small time windows, then we predict that the reconstruction error should increase if we perturb this coordination at that temporal resolution. One way to perturb the coordination among cells without destroying the firing statistics of each individual cell is to build artificial population responses from real ones as follows^41^: a long sequence of trials of population responses are generated, and artificial population responses are built by shuffling neural responses across trials while keeping neuron identity intact. In this way, artificial population responses are formed by responses of neurons that have not been observed together in the same trial. As predicted, when the coordination of responses were perturbed in this way, the reconstruction angular error was much larger in the trial-shuffled than in the original network (Fig. 5d, black and light blue). Moreover, when we shuffled bins of 100 ms within the same trial, while keeping neuron identity intact, the error also increased significantly (Fig. 5d, dark blue).

There is an interesting difference in the way the error decays over time when comparing trial-shuffled and bin-shuffled errors. This difference emerges because, as shown in Fig. 5c, the error on average decays with time, indicating that the network response is still in the transient period before stabilization. If trials are shuffled while maintaining neuron identity, the resulting populations of neurons are initially in rather different states because of the difference in initial conditions, causing the error to be comparatively larger at the onset of the trial period. However, at later times in the trial, the network state becomes more similar across trials because it is closer to the steady-state, resulting in smaller errors at later times when shuffling trials. This time-dependent modulation of the error does not arise when shuffling bins within a trial, because mixing early with later bins in the trial causes the error to be indistinguishable over time within a trial. Overall, the above analysis demonstrates that, at finite temporal resolution, firing rate covariations rather than precise spike timing, are responsible for encoding the stimulus and the underlying causes.

### Comparison with other spiking networks for related causal inference problems

Our spiking network is related, at the generative, algorithmic and implementation levels, to other neuronal networks for causal inference. At the generative level, our networks assume that there are a collection of latent causes weighted by non-negative numbers, representing both the presence and the intensity of the cause. This is identical to the assumption underlying non-negative matrix factorization (NMF) and the rate-based networks that have in the past been proposed for this problem^21^. In contrast to networks called Boltzmann machines, we assume a graded presence of causes that is encoded by non-negative intensities, whereas Boltzmann machines only allow for binary states in the world (cause present or not present) that are represented by binary-state networks^5^. More recent spiking networks have included the possibility of representing both the presence and the intensity of a cause by adding a separate non-negative intensity variable for each binary-state variable^25^. One significant difference to these is that our spiking network encodes both the presence of a cause and its intensity in the same latent variable, rather than encoding presence and intensity separately.

At the algorithmic level, our spiking network delivers the most likely (MAP) estimate of the causes given the observation using fully deterministic dynamics. This full determinism contrasts with the way Boltzmann machines operate^5^: samples of the probability distribution of the causes given the observations are generated by stochastic dynamics of such networks. As a result, the network samples causes that are most consistent with the stimulus. Similarly, a large family of rate-based and spiking networks have been recently discussed that operate under probability sampling algorithms^17^,^24^,^25^. We have mentioned above that the spiking dynamics of our overcomplete networks can be understood as a form of random walk. Naively, this might seem to imply that spikes in such a network can be viewed as samples of a probability distribution. However, this conceptualization is not accurate in our system: in reality, spikes are better conceptualized as ‘votes’ in favor of a cause. Initially the dynamics is highly competitive, with potentially several neurons emitting votes to support their ‘cause’. Spike-votes tend to inhibit other neurons in a highly competitive dynamics, implementing dynamic explaining away. This form of hard-logic stands in contrast to the soft-logic frameworks above mentioned, and might allow realizing deterministic symbol manipulations that could be deemed closer to human reasoning.

At the implementation level, our network computes with spikes, while large parts of research addressing causal inference have focused on rate-based implementations. One reason why rate-based networks have been favored is because they simplify mathematical analysis, typically rendering analytical results. Spike-based networks, in contrast, typically lack a mathematical foundation that allows analytical results. The spiking networks that we have described in this paper can be considered an exception in this regard, as exact solutions for the steady state of spiking networks, to our knowledge, have not been known so far. Using a recently developed framework^31^, we have been able to show that integrate-and-fire cells with a specifically tuned architecture and global inhibition are able to solve exactly and efficiently a high-dimensional causal inference problem. Another advantage of using spikes rather than rates is that in rate-based networks the non-negativity of neurons’ firing rate is enforced by adding linear rectification to the input currents (Eq. (9)). The resulting firing rates (i.e., spike counts divided by time) in spiking networks are by definition non-negative, allowing for a natural neuronal implementation of non-negativity inequality constraints.

More recently, a spiking network for the tracking of complex time-varying signals has been developed^14^. This network is similar to ours in that it is spike-based, and therefore falls into the class of biophysically plausible networks to solve causal inference problems. As our network, it has the goal of minimizing the squared error between actual and reconstructed stimuli. Therefore, it is not surprising that signal-tracking networks^14^, spike-based networks for approximate quadratic programming^23^ and other previous rate-based network for quadratic programming problems^21^,^22^,^42^ share network architectures that are very similar to ours (Eqs. (19)–(20), 27). The main difference to our networks lies at the details of the algorithm employed. While signal-tracking networks and versions thereof^14^,^23^ use a greedy minimization algorithm that, spike by spike, tries to minimize the reconstruction error in short time windows, our networks use a global algorithm that aims at finding the minimum in the long run.

**Figure 6:**
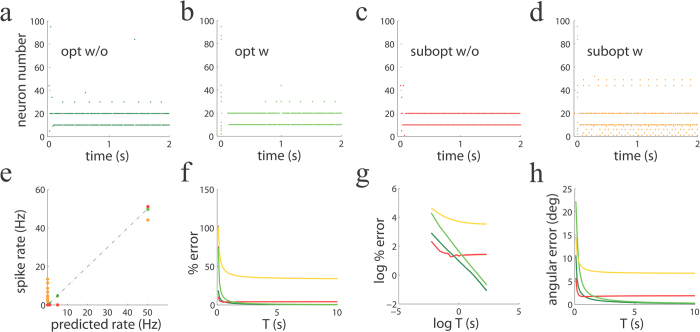
Performance of spiking networks for quadratic programming with optimal and suboptimal parameters. (**a**–**d**) Population activity patterns over time for optimal networks (no leak present) without (**a**, opt w/o) and with synaptic delays (**b**, opt w), and for suboptimal networks (leak present) without (**c**, subopt w/o) and with such delays (**d**, subopt w/o). (**e**) The optimal network matches the optimal solution. Observed firing rate of the spiking network vs. the rate predicted from a non-spiking algorithm for the same problem (rate-based network algorithm) for the networks displayed in panels (**a**–**d**). Color code is the same as the first row. Dark green dots are overlaid by light green dots, and therefore they are invisible. (**f**) Percentage error decays to zero as a function of the integration window *T* for optimal networks, but not for signal-tracking networks. (**g**) Log-log plot of the previous panel. The percentage error decays approximately as 1/*T* for the optimal network, and saturates for the signal-tracking network. (**h**) Angular error as a function of the integration window *T*.

This difference in algorithms is also reflected in a difference in the details of the implementation. First, signal-tracking networks mostly require instantaneous inhibition to operate efficiently. This is because inhibition is in charge of immediately suppressing the firing of other neurons once a particular neuron that represents the stimulus is active, to avoid over-representing the stimulus. Our networks, in contrast, do not require instantaneous inhibition: exponential synaptic kernels, or even delta-kernels with a delay, can be safely added to the dynamics and the network still operates efficiently (see Figs 2 and 6). This is made possible by considering the steady-state solution instead of a greedy, dynamic loss minimizer. Second, signal-tracking networks usually operate with leaky integrate-and-fire cells. Neurons in cortex feature such a leak, such that leaky networks can be considered more realistic than pure non-leaky networks. As we have shown in Fig. 3, the effect of leak in our network approximately implements L1-norm regularization. However, in general, the presence of leak makes deriving the steady-state solution of the system intractable, such that the exact computations underlying this solution remain elusive. Another upside of our non-leaky network is the integration of information without any information loss^31^, which is crucial for optimal functioning. The importance of using non-leaky networks for optimal computations have also been recently recognized in updated versions of signal-tracking networks^43^ and in networks for stable representation of memories^13^. In this work^43^, the authors have relaxed the instantaneous inhibition requirement of signal-tracking networks by using alpha function synapses. This makes their optimal network parameters depend on the shape of the synaptic kernel, while our optimal solution does not have to obey such dependency. Third and finally, the way L1 regularization is implemented in the network dynamics differs in signal-tracking networks and our networks: while signal-tracking networks implement L1 through an increase of both the spiking threshold and reset voltage, our networks implement L1 through global inhibition. While such a simultaneous increase of threshold and reset voltages can be realized by global inhibition when the network is leaky, this mapping becomes impossible for non-leaky networks. Furthermore, in some network implementations of signal-tracking networks^14^, the parameters values for L1 and L2 regularization substantially differ from those of our network (Eqs. (19)-(20), 27). In summary, focusing on the steady-state solution in non-leaky networks allowed us to solve the quadratic programming problem already considered in^23^ by spiking networks with significantly less constraints on synaptic kernels and a different implementation of L1 and L2-norm regularization.

Although the difference in parameters between signal-tracking and our causal inference spiking networks might seem minor, the two networks behave rather differently. This difference is to be expected because the networks implement different minimization algorithms, as mentioned above. To illustrate their different behavior, we chose delta-function kernels to allow a better comparison of the performance of these two types of networks. While signal-tracking networks have been found to show impressive performance in some tasks, here we compared their performance to our optimal networks in one of simple tasks that we have studied so far: mixture identification (Fig. 6a–d). For this case, the only architectural difference between the two types of networks is the presence or absence of leaks in the network. Additional differences are expected to arise when using regularization, which we do not consider in this simple example. When both our optimal network and signal-tracking networks have no synaptic delays, few neurons become active throughout the stimulation period (Fig. 6a,c). The population responses of the two networks are similar, but while our optimal network finds all components in the mixture (Fig. 6a; features number 10, 20, 30 and 40 weighted by different coefficients), the signal-tracking network tends to miss weak features that were presented in the stimulus (Fig. 6c; see the lack of activity of neurons 30 and 40). In the presence of synaptic delays, the optimal network undergoes a transient with many neurons firing initially, followed by a silence period that develops into a sparse population response where only neurons that fully identify the mixture are active (Fig. 6b). With synaptic delays, the signal-tracking network also features a transient period, but the final response is denser that in our network (Fig. 6d vs. b). The optimal network without or with delays finds the exact mixture of features that was used as input (Fig. 6e, green dots), but the suboptimal, signal-tracking network does not find the optimal solution (Fig. 6e, red and orange dots). Interestingly, we have also found that, despite slower convergence, our network still finds the optimal solution when the delay duration is significantly increased (e.g. 10 ms, v.s. the 2 ms used in Fig. 6). When the percentage reconstruction errors are compared across networks and conditions, we found that for optimal networks the error decayed over time approximately as 1/*T*, while the error saturated for the signal-tracking network (Fig. 6f,g). Similar results were observed when the reconstruction angular error was used (Fig. 6h).

At last we would like to re-emphasize that the above comparison is not meant to demonstrate that our networks will out-perform signal-tracking networks in all tasks. Its only purpose was to show that the deceptively small difference in network architecture between the two types of networks has significant consequences on their performance.

## Discussion

A spiking network that performs causal inference over a probabilistic domain has been described and analyzed. We have demonstrated that a selectively tuned network of integrate-and-fire neurons can deliver the set of most likely causes given a noisy observation of a linear combination thereof with non-negative coefficients. This problem involves a high-dimensional quadratic optimization with non-negativity inequality constraints that cannot be solved by linear networks. We have shown that our networks find the most likely causes using dynamic, spike-based explaining away by suppressing irrelevant causes when the observation can already be explained. The network design is remarkably straight-forward: network dynamics is based on linear synapses with realistic time scales, and uses the neurons’ spike-generating mechanism and reset as the only non-linearities. With this straight-forward hardware the spiking network can discriminate a cause among a multitude of other similar causes in just a few spikes. When confronted with a complex mixture of causes, the network can exactly and efficiently determine all the components of the mixture. The network is robust against internal and external sources of variability, as well as against connectivity mistuning. Information about the causes is encoded in the slow covariations of the firing rate of individual neurons, not in the individual firing rates separately or in the precise spike timing.

In perceptual and cognitive psychology, ‘explaining away’ is a hallmark of perceptual Bayesian inference and causal reasoning^2^,^3^ (see [5,26] for previous network implementations). Consider the example of trying to establish the cause for, one morning, observing a wet pavement. Potential causes could be that it rained overnight, or that a gardener has recently watered a park nearby. You look at the park, and see that there is a hose. Then, you will immediately imagine that it was a gardener who caused the pavement to be wet, discounting evidence for the possibility that it rained overnight. Therefore ‘gardener’ explains away the observations ‘wet pavement’ and ‘hose’. Our networks perform these type of computations with remarkable ease: a large set of causes dynamically compete for dominance through spike-based recurrent inhibition until a subset of them best explain the observation and suppress all other causes, resulting in the emergence of dynamic, spike-based explaining away. But how could a network like the one we have described in practice solve problems like the one just mentioned above? One can conceive a two-neuron network where the two causes, gardener and rain, are represented by features (1, 1) and (1, 0), respectively. Then an ambiguous input to the network representing wet pavement can be given to the network, represented by vector (2, 1). This input vector causes the two neurons to be active with equal firing rates, such that the network represents both gardener and rain. However, if the input to the network changes to (2, 2) to represent the additional observation hose, then the neuron encoding gardener will remain the only active neuron, explaining away rain. Therefore, the sum of observations does not result in a sum of causes; instead, it results in suppression of explained away causes.

So far we have assumed in our framework that the features have already been learned. In many relevant conditions, however, the features still need to be learned. Non-local learning rules for similar problems have been derived before and are applicable to rate-based implementations^21^. Local learning rules for linear problems have recently derived, and result in a combination of feed-forward Hebbian learning and anti-Hebbian learning that mediates the competition between the encoding neurons^44^ in rate-based networks. How to extend these learning frameworks to spike-based networks is still unclear, as it would require any form of communication, including learning, to be spike-based. First steps in this direction have already been performed^45^ and it might be possible that a comparable approach would also work for the particular problem we are considering. Another open problem is how task learning can be efficiently performed in spiking networks that learn prior distributions over the presence and intensity of causes. Our networks have partial flexibility to learn such priors, as both the reset voltage and the strength of global inhibition represent aspects of the probability of observing causes, but the plasticity rules that might govern learning in such cases remain to be investigated.

An important contribution of our work is to study the impact of spiking variability on network performance. Neurons in cortex fire in a variable way to repeated presentations to the same stimulus^33^,^34^ and this variability is correlated across cells^46^,^47^. Although variability appears to be harmful, especially when seen at the single-cell level, a recent study has shown that it does not limit sensory information^31^. First, the results presented here confirm those findings, as we have found that our networks encode sensory variables with high accuracy despite large amounts of variability (Fig. 4a). Second, and perhaps more importantly, we have gone one step further by showing that large mistuning in the connectivity matrix did not substantially limit network function, as long as the network was dynamically stable (Fig. 4d). Previous work has demonstrated that neuronal networks are robust against noise and mistuning of network parameters^13^,^14^, and our network reproduces this observation. This result, which holds even when mistuning was large, is by no means obvious as a mistuned network implements explaining away only in a loose manner. This observation suggests that explaining away might turn out to be a canonical computation that can be robustly implemented in a large repertoire of spiking networks.

Finally, it is important to highlight that high-dimensional causal inference requires a multi-dimensional network with many neurons that represent many potential causes. This fact precludes simplification of the network dynamics using standard mean-field techniques^48^ or other dimensionality reduction techniques, and warns research aiming at oversimplifying theoretical and experimentally measured neuronal dynamics to a few dimensions. The relevance of our theoretical network analysis consists in part in being able to solve analytically a large recurrent spike-based network with realistic synaptic dynamics. This allowed us to build the exact objective function of the system and map it to a causal inference problem. If we had reduced our spiking network to a small number of dimensions in neuronal space, we would have not been able to understand phenomena such as dynamic, spike-based explaining away in our networks. All in all, although additional realism needs to be added to the neuronal dynamics and harder problems need to be addressed, our results represent a concise example of how biophysically plausible spiking neuronal networks can perform exactly hard causal inference problems.

## Methods

### Simulations and numerical procedures

Simulations were performed using custom C code. Simulations were typically run for 100–1000 s with a one-step Euler method and a time step of 0.01 ms. We typically used exponential synaptic kernels *k*_*ij*_(*t*) (*i* ≠ *j*),


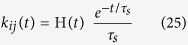


where H(*t*) is the Heaviside step function. We typically used *τ*_*s*_ = 5 ms. We also used delta-functions (*τ*_*s*_ = 0 ms, instantaneous inhibition) in some simulations.

#### Parameters

Parameters for simulations are specified in Table 1. In Fig. 2 we used the following feature and input vectors. Entries of the feature vectors were independently and identically distributed (i.i.d.) uniformly in the interval [0, 1], followed by normalization to one, 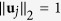. The connectivity matrix in the spiking network was generated using these feature vectors using Eq. (19), and hence was full rank. While network connectivity was kept intact in the three tasks, we chose the input vector differently for each task. In the discrimination task (Fig. 2, first row), the input vector was 50 times the 10^*th*^ feature vector. In the mixture identification task (second row), the input vector was 50 times the 10^*th*^ feature vector plus a background consisting of a sum of the remaining feature vectors with amplitudes i.i.d. uniformly in the interval [0, 10]. The background was constant throughout the trial. In the approximation task (third row), the first component of the input vector took value 1000 while the remaining components were set to zero. This vector lies with probability one outside the conic hull for the feature vectors generated as described above, and therefore it cannot be exactly expressed as a linear combination of the feature vectors weighted by non-negative coefficients.

In Fig. 3 we used a basis of feature vectors with entries i.i.d. following a uniform distribution in the interval [−0.5, 0.5] followed by normalization to one. We used *M* = 10 and *N* = 100 to create an overcomplete basis and to better study the effects of regularization on neuronal activity.

In Fig. 4, the *j*^*th*^ feature vector has component *i* equal to 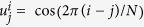. Because the feature vectors correspond to a basis of shifted cosines, the feature vectors effectively form a highly overcomplete basis. Hence the connectivity matrix obtained from these features vectors using Eq. (19) is low rank. The input vector was 50 times the 10^*th*^ feature vector. The optimal connectivity matrix was corrupted by frozen noise by adding an i.i.d. component to each entry uniformly distributed in the interval [−0.2, 0] (brown line in Fig. 4d). As feature vectors are normalized to one, the perturbation on the connectivity matrix was substantial, corresponding on average to a 75% change in the entry values from the optimal values.

For Fig. 5 we took the optimally tuned network of Fig. 4 and added i.i.d. (time-varying) white noise to each neuron in the network with variances 0, 0.01 and 0.3.

In Fig. 6 (panels a,b) we used a basis of feature vectors with entries i.i.d. following a uniform distribution in the interval [0, 1], followed by normalization to one, as in Fig. 2. The input vector was a linear combination of features 10, 20, 30 and 40 with coefficients 50, 50, 5 and 1, respectively. No background was added. Specifically in this simulations, we used delta-functions as synaptic kernels (*τ*_*s*_ = 0), with zero or 2 ms delays (in all other simulations exponential kernels were used). The signal-tracking network in Fig. 6 (panels c,d) had exactly the same architecture and synaptic kernels as the optimal one but neurons were LIF with reset voltage *H* = −0.5 and spiking threshold Θ = 0.5 (the same values can be chosen also for the non LIF network, and the results do not change because only the different between threshold and reset voltages matters).

Reconstruction errors were averaged across trials with different initial conditions (*n* = 200). Initial voltages for the neurons were randomly and uniformly sampled in the interval from reset to threshold voltages. The angular error is defined as the angle, averaged over trials, between the input vector (e.g. ***μ*** ∝ **u**_10_) and the reconstructed input vector, U**r** (Eq. (1)), where **r** are the estimated rates from the spiking network computed in time windows of size *T*. Fixed or varying sizes of the window *T* are used depending on the simulation and are indicated in each figure caption. When the term integration window is used, the origin of the time window is always at time zero. When the error is plotted over time, a moving window of fixed size is used. The (percentage) error is defined as 100 times the Euclidean distance, averaged over trials, between the input vector (***μ***) and the reconstructed input vector, *U***r** (Eq. (1)) (where **r** are the estimated rates from the spiking network computed in windows of size *T*, chosen as before) divided by the (Euclidean) length of the input vector. Error bars on the errors correspond to s.e.m. (*n* = 200).

## Additional Information

**How to cite this article**: Moreno-Bote, R. and Drugowitsch, J. Causal Inference and Explaining Away in a Spiking Network. *Sci. Rep.* **5**, 17531; doi: 10.1038/srep17531 (2015).

## Figures and Tables

**Table 1:** Parameter values.

Parameter	Value	Description
*N*	100	number of neurons, and number of features
*M*	100, 10 (Fig. 3), 2 (Fig. 4)	dimensionality of input vector
*τ*_*m*_	∞, 50 ms (Fig. 3), 20 ms (Fig. 6c,d)	membrane time constant
*τ*_*s*_	5 ms, 0 ms (Fig. 6; 0 or 2 ms delays)	synaptic time constant
Θ	1, 0.5 (Fig. 6c,d)	threshold voltage
*H*	0, −0.5 (Fig. 6c,d)	reset voltage
*α*	0, 10 (Fig. 3)	L1 regularization coefficient
*β*	0, 0.5 (Fig. 3)	L2 regularization coefficient
**U**	*M* × *N* matrix	feature vector matrix
***μ***	*M* × 1 vector	input vector

Default values are shown first. Figures where alternative values are used are indicated. None of the results depend critically on the exact values of the parameters.
